# Advancing the implementation and sustainment of medication assisted treatment for opioid use disorders in prisons and jails

**DOI:** 10.1186/s40352-019-0100-2

**Published:** 2019-12-12

**Authors:** Warren J. Ferguson, Joan Johnston, Jennifer G. Clarke, Peter J. Koutoujian, Kathleen Maurer, Colleen Gallagher, Julie White, Dyana Nickl, Faye S. Taxman

**Affiliations:** 1 Department of Family Medicine and Community Health, University of Massachusetts Medical School, 55 Lake Avenue North, Worcester, MA 01655 USA; 2 St. Vincent Hospital, Worcester, MA 01608 USA; 3 Rhode Island Department of Corrections, 40 Howard Ave, Cranston, RI 02920 USA; 4 Sheriff, Middlesex County, 400 Mystic Ave, 4th Fl., Medford, MA 02155 USA; 5 Connecticut Department of Correction, 24 Wolcott Hill Rd., Wethersfield, CT 06109 USA; 6 Health and Addiction Services, Connecticut Department of Correction, 24 Wolcott Hill Rd, Wethersfield, CT 06109 USA; 7 University Correctional health Care, Bates Bldg, 2nd Fl, New Jersey Department of Corrections, Trenton, NJ 08625-0863 USA; 8 Health and Criminal Justice Program, University of Massachusetts Medical School, 333 South St., Shrewsbury, MA 01545 USA; 9 Center for Advancing Correctional Excellence, 4087 University Drive, 4100, MSN6D3, Fairfax, VA 22030 USA

**Keywords:** MAT, Opioid, Criminal justice, Uptake, Implementation science

## Abstract

**Background:**

Opioid use disorder (OUD) is among the most prevalent medical condition experienced by incarcerated persons, yet medication assisted therapy (MAT) is uncommon. Four jail and prison systems partnered with researchers to document their adoption of MAT for incarcerated individuals with opioid use disorders (OUD) using their established treatment protocols. Employing the EPIS (Exploration, Planning, Implementation, and Sustainment) framework, programs report on systematic efforts to expand screening, treatment and provide linkage to community-based care upon release.

**Results:**

All four systems were engaged with implementation of MAT at the outset of the study. Thus, findings focus more on uptake and penetration as part of implementation and sustainment of medication treatment. The prevalence of OUD during any given month ranged from 28 to 65% of the population in the participating facilities. All programs developed consistent approaches to screen individuals at intake and provided care coordination with community treatment providers at the time of release. The proportion of individuals with OUD who received MAT ranged considerably from 9 to 61%. Despite efforts at all four sites to increase utilization of MAT, only one site achieved sustained growth in the proportion of individuals treated over the course of the project. Government leadership, dedicated funding and collaboration with community treatment providers were deemed essential to adoption of MAT during implementation phases. Facilitators for MAT included increases in staffing and staff training; group education on medication assisted therapies; use of data to drive change processes; coordination with other elements of the criminal justice system to expand care; and ongoing contact with individuals post-release to encourage continued treatment. Barriers included lack of funding and space and institutional design; challenges in changing the cultural perception of all approved treatments; excluding or discontinuing treatment based on patient factors, movement or transfer of individuals; and inability to sustain care coordination at the time of release.

**Conclusions:**

Adoption of evidence-based medication assisted therapies for OUD in prisons and jails can be accomplished but requires persistent effort to identify and overcome challenges and dedicated funding to sustain programs.

## Background

One in twenty-nine adults in the U.S. is estimated to have a lifetime experience of incarceration (Bureau of Justice Statistics, 2014), and this risk substantially increases for people of color and ethnic minorities (Pettit & Western, 2004). Estimates further indicate that 80% of all arrests can be traced to drug or alcohol use and associated lifestyles (The National Center on Addiction and Substance Abuse at Columbia University, 2010). Moreover, with 15% of incarcerated people having a serious mental illness, co-morbid illness is common; 30–40% have a chronic medical condition including infections spread through injection drug use.

The opioid epidemic in the United States has shed light on the impact of the crisis on criminal justice populations. The Center for Disease Control and Prevention reports that in 2016, individuals dying from overdose surpassed the numbers dying from AIDS at the height of that epidemic in 1994 (Centers for Disease Control and Prevention, 2001). The risk of overdose death in the first 2 weeks following release from prison or jail is particularly high, with one study estimating a relative risk of 129 times higher than individuals that do not experience incarceration (Binswanger et al., 2007). Findings from the 2017 legislatively mandated study of how to address the opioid epidemic in Massachusetts indicated persistent risk of overdose death post-release with 60% of individuals reported to have died from overdose were incarcerated in the year prior to their overdose (Bharel, 2017).

Despite the prevalence of substance use disorders, its downstream impact on justice involved persons and the constitutional right to health care for incarcerated persons (Estelle v. Gamble, 1976), few prisons and jails provide evidence-based substance use disorder treatments (Nunn et al., 2009; Taxman, Perdoni, & Harrison, 2007). This is particularly true for the use of medication-assisted therapy (MAT) for opioid use disorders despite a growing number of studies demonstrating the efficacy of the three FDA approved therapies: methadone, buprenorphine and naltrexone.

Given the high mortality associated with opioid use disorders, along with scientific evidence on the effectiveness of beginning MAT in jail/prison on continued treatment in the community (Kinlock, Gordon, Schwartz, Fitzgerald, & O’Grady, 2009), MAT treatment initiated during incarceration is gaining momentum. (National Sheriffs Association and NCCHC, 2018) It is critical to advance the science of implementing MAT in prisons and jails. A better understanding of the clinical and policy changes required to improve clinical outcomes is needed.

Implementation of evidence-based practices (EBPs) for substance use disorders have been rigorously studied along the continuum of criminal justice involvement including treatment-based diversion programs, jail- and prison-based programs and in community corrections. (Belenko, Hiller, & Hamilton, 2013; Taxman & Belenko, 2019) Yet, adoption of these EBPs has historically been low in the United States, and until recently, few have involved MAT. (Belenko et al., 2013) Typically, security concerns and cost have been intimated as the most important barriers to treatment. However, the stigma of substance use disorders and incarceration in the United States have also been intimated has important barriers to treatment. (Wakeman & Rich, 2018) Thus, as momentum increases to change policies toward adoption of MAT, implementation science methods are needed to understand the facilitators and barriers to providing MAT therapies in prisons and jails. Implementation science can facilitate a better understanding of the clinical and policy changes required to improve clinical outcomes.

Methadone, an agonist treatment therapy, was approved in 1972, and studies have demonstrated its efficacy when administered during incarceration, with more treated individuals at 12-months follow-up reporting less heroin use or engagement in injection drug use in the last 30 days (Brinkley-Rubenstein et al., 2018). Individuals taking methadone at the time of incarceration and who continued it through incarceration were more likely to be in treatment one-month post-release and less likely to be using injectable illicit drugs than those treated without agonists at the time of incarceration (Brinkley-Rubenstein et al., 2018). More recently, buprenorphine, a partial agonist approved by the FDA in 2002, was studied to determine outcomes when administration began in prison/jail or after release. Individuals receiving buprenorphine were more likely to remain in treatment at 6 months post-release and were less likely to be arrested in the past 30 days (Zaller et al., 2013). Naltrexone, a narcotic antagonist, given as a long-acting depot injection (Vivitrol, Alkermes) was approved by the FDA in 2010 for prevention of relapse among individuals with opioid dependence. It has been studied in persons under criminal justice supervision in community-based settings who desired to be in opioid-free treatment. An open-label randomized controlled study of naltrexone monthly injections for 6 months post-release vs. those not released on naltrexone demonstrated longer median time to relapse, lower rates of relapse, and higher rates of opioid-negative urine drug screens (Lee et al., 2016).

We report here on the implementation and sustainment of medication assisted treatment in two jails and two prison systems in New England who were at various points of implementing medication assisted therapies at the start of the study.

## Methods

### Participants

During the conceptualization and proposal for funding for this project, we approached four prison health care systems based in academic health centers across the country which agreed to participate in the study. However, once funding was procured, these four care systems were not engaged in substance use disorder treatment in prisons or jails and were unable to participate. Subsequently, we attempted to recruit several correctional health systems in the New England area based on our knowledge of planned or existing medication assisted treatment initiatives. Four systems agreed to participate. Two unified systems (combined prison and jail) in Rhode Island and Connecticut; and two Massachusetts-based jail systems, the Barnstable and Middlesex County Sheriff offices were recruited and enrolled. All signed memoranda of understanding to participate in the project.

### Design

A breakthrough collaborative methodology was adopted to engage project teams from each of the four systems. Breakthrough collaboratives have been employed as interventions to improve outcomes for myriad conditions, most frequently employing the Chronic Care Model (Chin, 2011; Chin et al., 2007; Coleman, Austin, Branch, & Wagner, 2009). The model calls for the development of change teams within each system focusing on the functions required to adopt evidence-based practices. Teams were encouraged to employ rapid cycle tests of change to refine implementation of evidence-based MAT practices for further penetration in the system with more clients or spread across the systems.

Content scaffolding for the implementation science study was guided by the model of evidence-based practice (EBP) implementation in public service sectors (Aarons, Hurlburt, & McCue Horwitz, 2011). This model describes four phases of implementation: exploration, preparation, implementation, and sustainment (EPIS) within the inner and outer contexts of influence on public sector systems.

### Learning sessions

The breakthrough collaborative model includes sessions to teach the knowledge and skills to advance medication assisted treatment in prisons and jails. Three learning sessions took place over the 18-month period of engagement. During session one, content experts for both medication-assisted treatment and implementation science presented the evidence base for medication assisted treatment and the model for implementation science study respectively. Teams were also oriented to expectations for engagement including data collection and monthly progress reports using the EPIS model for three distinct functions for a treatment program: opioid use disorder screening, treatment, and referral to community-based services at the time of release. Six months later, teams met again, and cross-team small groups were formed to map their best practices for these functions (screening, treatment, community-based treatment referral) as well as data collection systems. At the one-year mark, teams met again to report on their findings during presentations at the peer-reviewed Academic and Health Policy Conference on Correctional Health (Ferguson, 2018).

### Practice coach

A practice coach was available to teams to provide instruction on quality improvement and change management strategies. Site visits were conducted by the practice coach and when possible, the principal investigator, to learn more about the facility, current practices and progress to date via semi-structured interviews; and to provide advice when appropriate on strategies to accelerate change. Information collected at these meetings supplemented information from submitted progress reports to better inform details of facilitators and barriers to MAT implementation and sustainment.

### Data collection

Teams were asked to complete monthly data and narrative progress reports. Aggregated data reports focused on 1) the proportion of individuals screened for SUD, 2) the proportion screening positive for SUD, 3) the proportion diagnosed with opioid use disorder who were treated with MAT, 4) the proportion of those treated who received community referrals at the time of release and 5) the number of individuals who kept their initial outpatient referral appointments.

The monthly progress report utilized a template designed to describe details of adoption and adaptations to MAT practices, inclusive of facilitators and barriers influencing implementation and sustainment of the treatment program.

### Analysis

Run charts were created and analyzed rates of screening, treatment and community-based referral. The frequency of reporting and data content varied among participating sites based on local resources for data collection. In addition, it was difficult to compare results across sites as the populations of focus were different for agonist vs. antagonist treatment models as well as variations in program administration and community-based resources.

Progress reports and site visit meeting notes were analyzed by two authors (Ferguson, 2018) to catalogue best practices, innovations or barriers and to identify common themes that either facilitated or hampered adoption and expansion of evidence-based practices across the systems. These were categorized as outer/inner context influences and according to the EPIS framework.

## Results

### Site and medication assisted treatment descriptions

Table 1 describes the demographics of the four participating correctional systems involved in the study. The systems are very heterogeneous from the perspectives of their size, geographic setting, classification and movement of incarcerated persons and the geographic distribution of communities to which incarcerated persons return following release. The study began at different points in the adoption of medication assisted treatment and on the type of treatment being offered to incarcerated patients (agonist, antagonist or both) as well as the type of agonist treatment when applicable. The two Massachusetts jail systems offered antagonist treatment with depot injectable naltrexone (Vivitrol: Alkermes) exclusively while the two prison systems offered both agonist and antagonist treatment. Connecticut offered methadone as their agonist in addition to depot injectable naltrexone, while Rhode Island was the only participating system to offer both methadone and buprenorphine-naloxone along with depot injectable naltrexone.

**Table 1:** Characteristics of Correctional Systems studied

Correctional System	Jurisdiction	Jails/Prisons	Number of Facilities	Sites offering MAT^a^	Population
Middlesex County Jail and House of Correction	County	Jail/HOC^b^	1	1	1160
Barnstable County Jail	County	Jail/HOC	1	1	350
Rhode Island Department of Corrections	State	Jails and Prisons	6	6	3100
Connecticut Department of Corrections	State	Jails and Prisons	15	3	14,815

^a^*MAT* Medication-assisted treatment, ^b^*HOC* House of correction

### Quantitative findings

Reporting parameters were different depending on the nature of whether the primary treatment was agonist or antagonist. For the two sites offering agonist treatment, the focus was on evaluation of patients from the time of entry into the system while the two jail systems offering primarily antagonist treatment focused their efforts on engaging patients and initiating treatment in the 2 months pre-release. For 9 months, three of four sites reported quantitative data for screening, treatment and care coordination at the time of release. One site solely reported only the number of individuals treated from month to month.

Because of variation in the size of the facilities, the range of the populations admitted to facilities was large, 40 to 350 during any given month. By the third month of data collection, sites consistently screened 100% of all individuals for SUD at the time of intake and screening rates remained consistent for the duration of project reporting. The proportion of individuals screening positive for opioid use disorders was quite high and varied from month to month with a range of 27–65%. For those reporting on the rates of provision of medication assisted treatment over time, the range was again quite large, from 9 to 61% of those diagnosed with opioid use disorders during any given month of reporting. With respect to MAT treatment expansion over time, only one site demonstrated consistent growth in the number of patients treated over the course of the study.

For the two sites exclusively treating with Vivitrol prior to release, 100% of individuals were released with appointments to community-based providers post-release for the 9 months of data reporting, with show rates for those appointments varying from month to month, with a range of 35 to 100%, (mean = 65%). Due to aggregated data and small number of individuals receiving treatment, no specific trend line or statistical difference across sites could be calculated.

### Qualitative findings

**Fig. 1:**
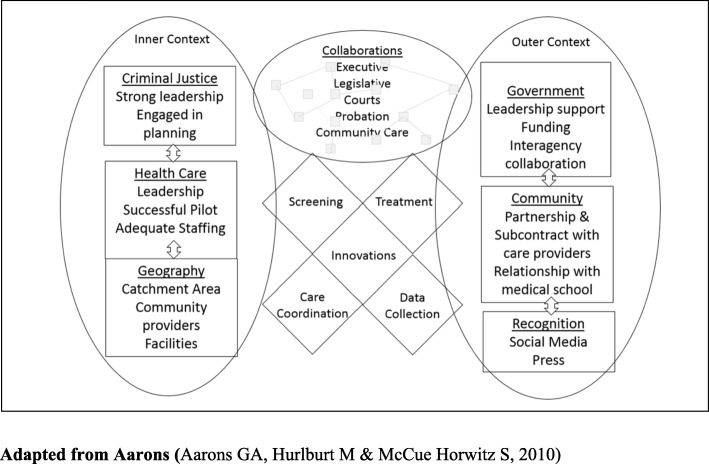
Outer and inner context influences for MAT. Legend: Adapted from Aarons (Aarons et al., 2011)

Outer and inner context influencers of implementation and sustainment of medication assisted treatment are depicted in the Fig. 1. While issues such as funding and staffing levels are important elements of most change efforts, a few deserve special mention given the intersection of health and criminal justice systems in the provision of care. Leadership was a critical driver for successful implementation, both from outer context (e.g. Governor, Legislature) and from inner context (e.g. Commissioner, Sheriff). All leaders demonstrated passion for improving the outcomes of the opioid crisis in their communities and their leadership was demonstrated by the commitment of their teams engaged in treatment. This was also critical for developing a shared mission between health care and security missions of the organizations. Community-based partnerships were also critical elements for success for both outer and inner contexts. All systems prioritized the importance of care coordination post-release. Interestingly, the challenge of establishing community-based treatment may have impeded increased rates of treatment in one site. Contracted services for the delivery of agonist medications on-site were observed to be an innovation to accelerate the spread of treatment from several perspectives.

To implement agonist treatment programs, existent staffing levels were deemed insufficient. Moreover, extensive training of existing staff would be required. Second, the process for obtaining a Drug Enforcement Agency (DEA) certification as a methadone treatment facility is expensive and can take up to a year. Thus, contracting with a community-based methadone provider solved both problems and provided an added benefit for seamless care coordination at the time of release to community based treatment programs operated by these contracted organizations.

Influences on the decision to offer both agonist and antagonist therapies as opposed to antagonist treatment only are important. Both in-system and government leadership influenced the types of therapy offered and expansion of treatment. At sites offering agonist treatment, a focus on fidelity to evidence-based therapies was an important influencer, with articulated belief to engage patients in the best choice of therapy in consideration of their health care issues. Where antagonist-only treatment was offered, security concern regarding diversion of agonist medications and the cost of medications were prominent. Of note, those sites offering agonists often had to overcome these concerns on the part of their security missions and health care staff to implement agonist treatments. An important factor influencing both health care and security staff regarding whether agonist treatment should be offered was alignment with government and institutional leaders’ priorities.

At the start of the programs, systems were engaged in different phases of medication assisted treatment, from planning to sustainment. Therefore, most findings focus on these steps in the EPIS model. While some operational elements were common for both agonist and antagonist treatment sites, agonist treatment focuses largely on treatment at the time of facility intake while antagonist treatment focuses more on pre-release treatment. Implementation facilitators and barriers identified through content analysis of progress reports and meeting notes are grouped according to screening, treatment, community care coordination and data collection in Table 2. Facilitators for adoption of medication assisted treatment included: funding; management of culture change; addition of staff and staff training; networking with other elements of criminal justice system (probation, courts) and community based treatment providers; spread of treatment to pre-trial and work release populations; developing effective data collection methods and the use of data to improve processes; organization of group visits for both education and care delivery; and strategies to keep individuals in treatment post-release. Data collection methods and systems were a challenge for some sites, requiring sites to create their own excel-type records. Some sites focused on new modules in electronic medical records and identified dedicated staff for data collection and analysis.

**Table 2:** Operational factors influencing MAT implementation and sustainment

Screening Aim: Employ an initial screen for opioid use disorder at intake for 100% of new admissions		
Category	Barriers:	Solutions and Innovations: (bold = innovation)
Facility	Space at intake not conducive to screening	**Self-administered screen with tablet at intake**
Culture and Change Management	Inconsistent screening due to custody vs. medical priorities	Aligned custody and medical leadership
Policy/Procedure	Lack of standardized procedure for screening and assessment	Develop a comprehensive screen to be completed by Day 2
Education	Lack of education on medication assisted treatment options and recovery treatment	**Video education at intake about treatment program**
Staffing & Training	Insufficient staff to screen consistently	Train interdisciplinary staff to screen; temporary increase in staffing during busy times
IT/EMR		**Use tablet technology for screening linked to EMR**
Treatment Aim: To offer system-approved treatment to all individuals diagnosed with opioid use disorder unless treatment is contraindicated		
Category	Barriers	Solutions and Innovations: (bold = innovation)
Culture & Change Management	Lack of buy-in from Security and Nursing; Judgement that patient is “poor candidate” for treatment or terminate treatment due to “bad behavior”; contraband concerns of custody	Alignment of custody and medical priorities through training and open dialogue policy to continue all FDA approved treatment at time of incarceration
Staffing	Medical services not 24/7; insufficient staff for treatment induction	Increase capacity to treat 24–7; add staff during peak days; contract with community-based provider to assist with treatment onsite; train staff to be flexible
Policy/Procedure	No standard process for treatment induction	Create comprehensive treatment procedures
Patient knowledge & education	At jails providing agonist treatment, many patients express lack of interest in treatment	Focus groups to explore lack of interest in treatment and group education visits to address concerns
Facility	Space not conducive to treatment	Site expansion; medication line customization; designated housing units for treatment
Contraindication	Medical conditions preclude treatment; e.g. liver disease; medication side effects intolerable	Provide alternative medication
Safety Concern/Procedure	Inmate movement and transfers	
Spread and expand treatment		Criminal justice collaborations: pre-trial, drug court, work release populations
Practice transformation		Add CBT; interdisciplinary team approach; structure improvement efforts into smaller functional work groups; treatment integrated into standard operating procedures
Community coordination for post-release care Aim: 100% of treated patients will receive an appointment for treatment at time of release and all appointments will be kept		
Category	Barriers	Solutions and Innovations
Community Access	Large geographic catchment for return to home post-release	Develop a community/county reentry council
Patient tracking	Data not available from community agency; lose patients to follow-up	Contract with community-based treatment provider for onsite treatment; identify liaison with community-based providers; recovery specialist or coach follows patient post-release; close coordination with courts and probation
Insurance	Lack of access to post-release treatment or transportation issues; lack of health insurance at time of release;	Work with state to suspend public insurance and reactivate at time of release; expand state Medicaid enrollment; work with community providers willing to provide ‘bridge’ services
Staffing	Insufficient staff for discharge planning	Develop follow-up process for patients released on treatment; Cross-train all discharge planners to coordinate post-release treatment; addition of recovery coaches; CMS waver for 30-day pre-release planning
Post-release programming		Aftercare group for released population on treatment; job placement in recovery friendly environment; open step-down unit run by prison or jail
Data collection systems: develop system for tracking patients screened with OUD, those treated and untreated as well as community referral tracking		
Category	Barriers	Solutions and Innovations
Data collection and reporting	Manual data collection with data entry in Excel; errors in secondary data entry; status revision requires repeated data input already entered	Fully integrated EMR with MAT assessment and treatment information and reporting capacity
Staffing	Limited staff for data collection and reporting	Peer navigators assist with intake and referral data entry
Culture and Change Management	Data collection and reporting not a priority	Prioritize value of data across public safety and coordinate with all agencies

Barriers to adoption and expansion of best practices included the amount and design of space; cultural barriers to adoption of SUD treatment by both clinical and security staff; security, medical or treatment motivation assessments that prevented access to treatment; movement and transfers between facilities; and large geographic catchments that precluded consistent post-release engagement into care. Location of facilities in states with expanded Medicaid provided opportunities to work with state Medicaid programs to suspend public health insurance coverage for covered individuals and efficient reactivation at the time of release. At times, arranging for access for community-based treatment follow-up was a barrier.

## Discussion

We partnered with four prison and jail systems using MAT to treat opioid use disorder who desired to sustain and/or expand the number of individuals in treatment during incarceration and to coordinate care post-release. The findings are observational and focused on the influences that facilitated or impeded implementation and sustainment of treatment as well as interventions employed to improve rates of screening, treatment and care coordination post-release.

The prevalence of OUD in housed populations was quite high, ranging from 27 to 65% in any given month. Leadership and collaboration between criminal justice and health care entities were critical to implementation and sustainment as was networking across all segments of the criminal justice system (courts, probation, jails and prisons). Addressing the high risks of overdose and death post-release were important motivators in all four systems. Screening and coordination of care post-release were improved over time. Only one system was successful in increasing the proportion and total number of individuals receiving treatment for OUD. Funding and insufficient numbers of staff were frequently noted as obstacles. At one facility, a treatment rate-limiting problem was care coordination with community-based providers due to limited capacity and the large number of counties to which individuals were returning home post-release. At one jail site, this was noted as a concern for consideration of expansion to include agonist treatment. The two systems offering agonist treatment had sufficient resources to contract with community-based treatment organizations to provide services onsite and to provide seamless coordination of care following release. This proved to be an important facilitator for increasing rates of treatment in one system over the course of the observational period.

The study has several limitations. First, it was conducted in four small states in one region of the country. While we set out to collect data from all four systems on rates of screening, treatment and care coordination post-release, lack of funding made data collection unsustainable for at least two systems. The challenges of conducting MAT research in criminal justice populations has been noted by others as well (Gordon, Kinlock, & Miller, 2011). Moreover, the population of focus for capturing data is time-dependent and treatment-dependent. For those patients receiving agonist treatment, the focus is on treatment at the time of entry into custody while the time prior to release is the treatment period of interest for sites proving antagonist-only MAT.

We believe that this study contributes new knowledge on implementation and sustainment of medication-assisted treatment which will be helpful to those systems initiating treatment efforts. Going forward, we hope to expand on this research by studying a new pilot program offering all approved medications for OUD. In July 2019, Massachusetts passed legislation establishing an MAT pilot program in partnership with seven Sheriffs’ offices and the Massachusetts Department of Correction. The three-year pilot funded by the Commonwealth will allow participating correctional facilities to offer all forms of MAT to provide post release navigation services for participants and includes a robust data collection component for policy analysis and long-term planning. (The 191st General Court of the Commonwealth of Massachusetts, 2018).

## Conclusions

Adoption of evidence-based medication assisted treatment for substance abuse disorders in prisons and jails can be accomplished when persistent and ongoing efforts to identify and overcome challenges are present. These findings should inform other correctional programs considering initiation or expansion of medication assisted treatment for opioid use disorder.

## Data Availability

All data generated during this study are included in this published article.

## Abbreviations

AIDS: Acquired Immune Deficiency Syndrome
EBP: Evidence Based Practice
EPIS: Exploration, Preparation, Implementation, Sustainment
FDA: Food and Drug Administration
MAT: Medication Assisted Therapy
OUD: Opioid Use Disorder
SUD: Substance Use Disorder
